# Enhancer of zeste homolog 2 (EZH2) expression is an independent prognostic factor in renal cell carcinoma

**DOI:** 10.1186/1471-2407-10-524

**Published:** 2010-10-04

**Authors:** Nina Wagener, Stephan Macher-Goeppinger, Maria Pritsch, Johannes Hüsing, Karin Hoppe-Seyler, Peter Schirmacher, Jesco Pfitzenmaier, Axel Haferkamp, Felix Hoppe-Seyler, Markus Hohenfellner

**Affiliations:** 1 German Cancer Research Center, Molecular Therapy of Virus-Associated Cancers (F065), Im Neuenheimer Feld 242, 69120 Heidelberg, Germany; 2 Department of Urology, University of Heidelberg, Im Neuenheimer Feld 110, 69120 Heidelberg, Germany; 3 Institute of Pathology, University of Heidelberg, Im Neuenheimer Feld 220/221, 69120 Heidelberg, Germany; 4 Department of Medical Biometry, University of Heidelberg, Im Neuenheimer Feld 305, 69120 Heidelberg, Germany; 5 Coordination Centre for Clinical Trials (KKS), University of Heidelberg, Voßstraße 2, 69115 Heidelberg, Germany; 6 Department of Urology, Evangelisches Krankenhaus Bielefeld, Schildescher Strasse 99, 33611 Bielefeld, Germany; 7 Department of Urology, University of Frankfurt am Main, Theodor-Stern-Kai 7, 60590 Frankfurt am Main, Germany

## Abstract

**Background:**

The *enhancer of zeste homolog 2 *(*EZH2*) gene exerts oncogene-like activities and its (over)expression has been linked to several human malignancies. Here, we studied a possible association between EZH2 expression and prognosis in patients with renal cell carcinoma (RCC).

**Methods:**

EZH2 protein expression in RCC specimens was analyzed by immunohistochemistry using a tissue microarray (TMA) containing RCC tumor tissue and corresponding normal tissue samples of 520 patients. For immunohistochemical assessment of EZH2 expression, nuclear staining quantity was evaluated using a semiquantitative score. The effect of EZH2 expression on cancer specific survival (CSS) was assessed by univariate and multivariate Cox regression analyses.

**Results:**

During follow-up, 147 patients (28%) had died of their disease, median follow-up of patients still alive was 6.0 years (range 0-16.1 years). EZH2 nuclear staining was present in tumor cores of 411 (79%) patients. A multivariate Cox regression analysis revealed that high nuclear EZH2 expression was an independent predictor of poor CSS (> 25-50% vs. 0%: HR 2.72, p = 0.025) in patients suffering from non-metastatic RCC. Apart from high nuclear EZH2 expression, tumor stage and Fuhrman's grading emerged as significant prognostic markers. In metastatic disease, nuclear EZH2 expression and histopathological subtype were independent predictive parameters of poor CSS (EZH2: 1-5%: HR 2.63, p = 0.043, >5-25%: HR 3.35, p = 0.013, >25%-50%: HR 4.92, p = 0.003, all compared to 0%: HR 0.36, p = 0.025, respectively).

**Conclusions:**

This study defines EZH2 as a powerful independent unfavourable prognostic marker of CSS in patients with metastatic and non-metastatic RCC.

## Background

Renal cell carcinoma (RCC) is estimated to account for more than 57000 new cases and 13000 cancer-related deaths in the United States in 2009, making it the second most lethal of all urological cancers [1]. Defects in von Hippel-Lindau (VHL) tumor suppressor gene function appears to be one key event in clear cell RCC, in both hereditary and sporadic cases [2]. However, the variable clinical picture of the resulting neoplasms is likely to be strongly determined by the complex interplay of additional cellular alterations, among which the role of epigenetic modulation of gene expression is becoming more and more acknowledged [2].

The *enhancer of zeste homolog 2 *(*EZH2*) gene encodes a polycomb group (PcG) protein which acts as a histone methyltransferase [3,4] and also may control DNA methylation [5]. There is accumulating experimental evidence that *EZH2 *can contribute to the deregulation of cellular growth as a *bona fide *oncogene. Overexpression of *EZH2 *conferred cellular growth advantage *in vitro *[6,7], promoted invasion [7], and exhibited oncogenic properties in nude mice [8]. *Vice versa*, inhibition of *EZH2 *expression by antisense constructs or RNA interference (RNAi) resulted in growth inhibition of cancer cells [9,10], and induced anoikis in circulating prostate carcinoma precursor cells [11] or apoptotic cell death in breast cancer cells [12]. We recently found that inhibition of endogenous *EZH2 *expression in RCC cell lines by RNAi was linked to reduced proliferation and increased apoptosis in RCC [13] and cervical carcinoma cells [14].

Notably, EZH2 may serve as a novel marker with potential for clinical oncology. EZH2 expression was linked to an increased risk for breast cancer development in females [15,16], suggesting that EZH2 detection could have diagnostic value for this cancer form. Furthermore, in both prostate [9,17] and breast cancer [7,17], EZH2 expression was associated to more aggressive tumor subgroups, indicating that EZH2 expression may also serve as a novel prognostic marker.

In view of its growth-promoting activities in RCC cell lines [13], we here investigated the potential of EZH2 to serve as a prognostic marker for RCC. EZH2 protein expression was analyzed in primary RCC specimens and in corresponding non-tumorous tissue, using a tissue microarray (TMA) encompassing tissue cores of 520 patients. In addition, we investigated the association of EZH2 expression with cancer specific survival (CSS) in univariate and multivariate Cox regression analyses. We show that EZH2 is significantly overexpressed in primary RCC, when compared with histologically normal renal tissue. Moreover, high nuclear EZH2 expression in non-metastatic disease, and nuclear EZH2 expression in metastatic disease are unfavourable independent predictive parameters of CSS. The concordance probability of the Cox regression models including EZH2 was higher compared to models excluding EZH2, for both metastatic and non-metastatic disease. We conclude that EZH2 is a powerful and independent predictor of RCC-related death, which can add to the development of a modified risk stratification system.

## Methods

### Patients

Clinical data of patients (n = 768) with RCC who underwent radical nephrectomy or nephron-sparing surgery at the Department of Urology, University of Heidelberg, between 1990 and 2005 and had no other malignant tumor before or within one month after surgery were entered into a prospective database. Tumor stage was classified according to the tumor node metastasis staging system of 2002, tumors were graded on the basis the Fuhrman four-tiered nuclear grading system. In patients with metastases, the Motzer criteria (haemoglobin, corrected calcium, and Karnofsky performance scale) [18] were evaluated and patients categorized to one of the following risk groups: low, intermediate or high risk. Patients were prospectively evaluated every 3 months for the first 2 years after surgery, every 6 months for the next 3 years, and yearly thereafter (chest x-ray or thoracic CT scan; abdominal sonography or CT scan or MRI; serum chemistry). No adjuvant therapy was administered after radical surgery. Patients with metastases, a Karnofsky severity rating ≥ 80, and with no medical contraindications received palliative Interferon-alpha- and Interleukin-2-based immunotherapy. No tyrosine kinase inhibitors have been given.

The work was covered by a votum of the ethical committee of the University of Heidelberg No. 206/2005. Informed and/or written consent was obtained from each patient.

### Tissue micro arrays

Tissue samples of all 768 patients included in the prospective clinical database were obtained from the Tissue Bank of the National Center for Tumor Diseases (NCT) Heidelberg and included in a TMA, containing 768 primary tumor and corresponding normal tissue samples, as previously described [19]. Briefly, representative tissue blocks were selected as donor blocks for the TMA. Sections were cut from each donor block and stained with H&E. Then, a morphologically representative region was chosen from each of the RCC and normal renal tissue samples. Two cylindrical core tissue specimen per tumor block (diameter, 0.6 mm) were punched from these regions and arrayed into the recipient paraffin block using a semiautomatic system (Beecher Instruments, Silver Spring, Maryland, USA). In total, 16 tissue array blocks were generated, each containing up to 200 core tissue specimens, matching 50 patients per array.

### Immunohistochemistry

The TMA slides were dewaxed and rehydrated using xylene and a series of graded alcohols, followed by heat induced antigen retrieval using a target retrieval solution (S2031, DakoCytomation, Glostrup, Denmark) in a pressure cooker for 10 min. Immunohistochemical staining was performed on an automated staining system (Techmate 500, DakoCytomation) with a mouse monoclonal anti-EZH2 antibody (1:25, BD Transduction Laboratories, Franklin Lakes, NJ, USA) for 45 min [15]. An avidin-biotin-complex peroxidase technique using aminoethylcarbazole for visualisation and hematoxylin for counterstaining was applied. In accordance with the manufacturers' instructions, the following solutions were used: ChemMate Detection Kit (K5003, DakoCytomation), ChemMate Buffer Kit (K5006, DakoCytomation), and, for reduction of non-specific avidin/biotin-related staining, the Avidin/Biotin Blocking Kit (SP-2001, Vector Laboratories, Burlingame, USA). Sections were thoroughly washed, glass covered, and analysed by light microscopy (Olympus Vanox-T, Hamburg, Germany), using a magnification of up to × 400. Specificity controls of the anti-EZH2 antibody included iliac lymph node metastases of prostate carcinoma [17] showing typical staining pattern. Moreover, reactive infiltrating lymphocytes, which express detectable amounts of EZH2 protein [20], served as additional internal positive controls. As a negative control for the immunohistochemical staining procedure, the primary antibody was omitted, with all other experimental conditions kept constant. For immunohistochemical assessment of EZH2 expression, frequency of nuclear staining was evaluated using a semiquantitative score: 0 = no expression; 1 = positivity in 1 to 5% = low expression; 2 = positivity in >5 to 25% = intermediate expression; 3 = positivity in >25 to 50% = high expression; and 4 = positivity in more than 50% = very high expression. The arrays were independently scored by two researcher blinded for patient outcomes. For the few instances of discrepant scoring, a consensus score was determined.

### Study design

The study has been conducted on the REporting recommendations for tumour MARKer prognostic studies (REMARK) of the NCI-EORTC Working Group on Cancer Diagnostics [21]. A retrospective study design was chosen, based on a prospective clinical database. Data analysis commenced in June 2006. Median follow-up of patients still alive was 6.0 years (range 0-16.1 years).

Patients' CSS was calculated from the date of renal surgery. The survival endpoint was the date of last follow-up or death. Kaplan-Meier estimates were used to describe survival rates including pointwise asymptotic 95%-confidence intervals using Greenwood's formula for standard error. Patients with proven tumor independent death were censored. Furthermore, assuming independence of the occurrence of RCC and other primary tumors in the same patient, patient survival was censored at the time of the occurrence of a second malignoma.

The following clinical and pathological features were studied for their prognostic relevance on long term survival of RCC patients: Age (≥65 years vs. <65 years), sex (male vs. female), performance status (Karnofsky severity rating <80 vs. ≥80), tumor stage (Stage II, III, IV vs. I), Fuhrman's grade (grade 2, 3/4 vs. 1), histopathological subtype (clear cell RCC vs. other types), nuclear EZH2 expression (1-5%, >5-25%, >25-50%, >50% vs. 0%).

### Statistical analysis methods

Association between important prognostic factors and EZH2 levels was evaluated by Fisher's exact test, in case of large contingency tables the Monte Carlo Simulation was used.

For the evaluation of prognostic factors the study population was divided into subgroups with and without metastatic disease.

No data driven combination of adjacent categories related to EZH2 expression was carried out to retain the confirmatory nature of the evaluation of EZH2.

Univariate and multivariate analyses of prognostic factors were carried out within the Cox proportional hazards model using complete cases analysis.

For each prognostic factor the hazard ratio in the univariate analysis and the adjusted hazard ratio in the multivariate analysis are given, including the 95% confidence interval. A p-value < 0.05 was considered significant. For further description of the predictive value of EZH2 the concordance probability [22] of the Cox models including EZH2 were calculated and compared to the models excluding EZH2 but retaining all other variables.

The statistical analysis system SAS, Version 9.2 for Windows (SAS Institute Inc., Cary, NC, USA), the R package, Version 2.8.0 (R Foundation for Statistical Computing, Vienna, Austria), and StatXact, Version 8.0.0 (Cytel Inc., Cambridge, CA, USA) were used for the analyses. For the calculation of the concordance probability, the CPE package offered by Mo, Goenen and Heller was used with the R programming language.

## Results

In order to identify prognostic markers for RCC in a large cohort of patients, a TMA was constructed which contained RCC tumor tissue and corresponding normal renal tissue samples from 768 patients. Expression of EZH2 was analyzed by immunohistochemistry using a mouse monoclonal anti-EZH2 antibody [15]. Altogether, 520 cases (non-metastatic disease n = 433, metastatic disease n = 87) were scored for expression of EZH2. The remaining cases with insufficient tumor tissue or fixation artefacts were excluded from further analyses.

**Figure 1:**
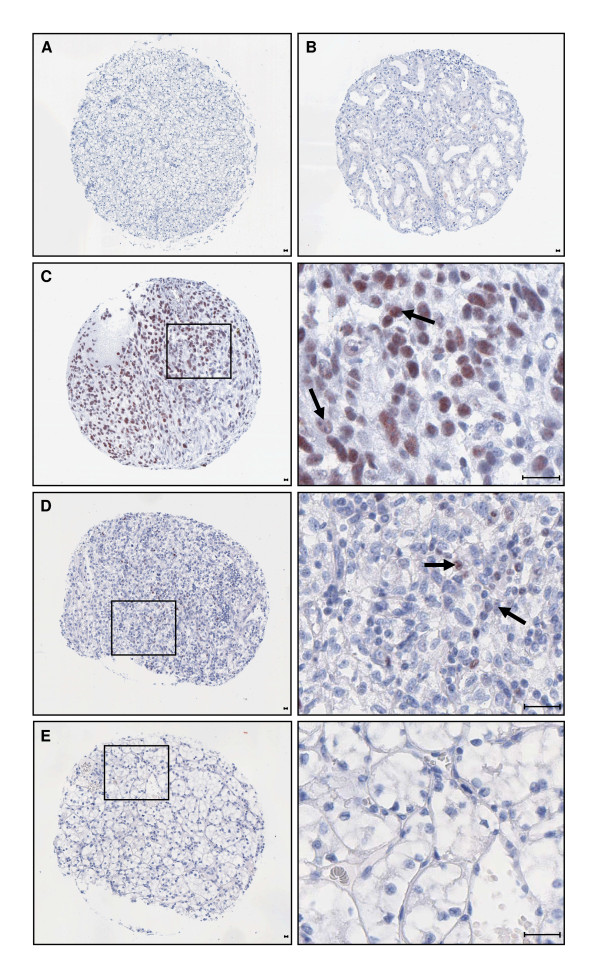
**EZH2 protein expression in renal cell carcinoma (RCC) and corresponding normal tissue**. **A** Negative control: RCC tissue specimen (clear cell carcinoma), the anti-EZH2 antibody was omitted. **B **Normal adult kidney tissue, adjacent to the RCC tumor tissue shown in C. **C **Overview (left panel) and higher resolution (right panel) of the boxed area of an RCC sample (clear cell carcinoma), showing very high (> 50%) nuclear EZH2 expression (arrows). **D **Overview (left panel) and higher resolution (right panel) of an RCC sample (clear cell carcinoma) showing >5-25% nuclear EZH2 expression (arrows). **E **Overview (left panel) and higher resolution of an RCC sample (clear cell carcinoma) exhibiting 0% nuclear EZH2 staining. Scale bars, 5 μm.

Typical examples are depicted in Figure 1. Negative controls showed no immunohistochemical staining (Figure 1A). Non-tumorous kidney tissue was mainly negative for EZH2 expression (Figure 1B), only sporadically proximal and distal tubular epithelial cells and infiltrating lymphocytes, if present in the tissue sample, stained positive for EZH2. In contrast, EZH2 protein expression was readily detected in 411 (79%) of the cancerous tissues, typically showing nuclear staining (Figures 1C and 1D), without predominately staining of the central or peripheral zone of tumor. Ten (2%) cases exhibited >50% EZH2-positive nuclei (Figure 1C), 36 (7%) >25-50% EZH2-positive nuclei, 99 (19%) >5-25% EZH2-positive nuclei (Figure 1D), and 266 (51%) 1-5% EZH2-positive nuclei. In contrast, 109 samples (21%) did not exhibit EZH2 nuclear staining in the tumorous tissue (Figure 1E).

Next, the survival of patients with RCC was calculated from the time of renal surgery. Until June 2006, 147 patients (28%) had died of their disease. The CSS rate at 1 year and at 5 years after surgery for the whole cohort of patients was 88.4% (95 CI 86.1%-90.7%) and 70.8% (95 CI 67.3%-74.4%), respectively. Clinical and pathological features of patients are summarized in Table 1.

**Table 1:** Summary of clinical and pathological features (study population n = 520)

Feature		Number of cases	% of cases
**Sex**			
	Male	320	61.5
	Female	200	38.5
**Age at surgery**			
	< 65 years	295	56.7
	≥ 65 years	225	43.3
**Karnofsky perfomance status scale**			
	≥ 80%	476	91.5
	< 80%	44	8.5
**Tumor extent (Robson)**			
	Stage I	310	59.6
	Stage II	37	7.1
	Stage III	83	16.0
	Stage IV	90	17.3
**Regional lymph node metastasis**			
	N0/pN0	481	92.5
	pN+	39	7.5
**Distant metastasis**			
	M0	433	83.3
	M+	87	16.7
**Fuhrman grade**			
	G1/G2	417	80.2
	G3/G4	98	18.8
	Unclassified	5	1.0
**Histopathological subtype**			
	Clear cell RCC	422	81.1
	Papillary (chromophilic) RCC	55	10.6
	Chromophobe RCC	23	4.4
	Duct bellini	3	0.6
	Unclassified RCC	17	3.3

Patients with advanced tumor stages (3 and 4), with cancer infiltrated lymph nodes, distant metastases and/or a high Fuhrman's grading (grade 3/4) were found to have significantly more often a higher percentage of EZH2 nuclear staining when compared to patients with localized tumor disease or low Fuhrman's grading (grade 1/2). Moreover, nuclear EZH2 expression and the Motzer criteria were found to be independent parameters, whereas a dependency was seen between different histopathological subtypes and nuclear EZH2 expression (Table 2).

**Table 2:** Correlation of nuclear EZH2 expression with clinicopathological characteristics

		Nuclear EZH2 expression										
		0%		1-5%		> 5-25%		> 25-50%		> 50%		
		n	%	n	%	n	%	n	%	n	%	*p*
**Tumor extent (Robson)**												***0.0071***
	Stage I	65	60	167	63	61	62	14	39	3	30	
	Stage II	9	8	24	9	2	2	2	6	0	0	
	Stage III	20	18	38	14	15	15	8	22	2	20	
	Stage IV	15	14	37	14	21	21	12	33	5	50	
**Sex**												*0.984*
	Male	67	61	161	61	63	64	23	64	6	60	
	Female	42	39	105	39	36	36	13	36	4	40	
**Age at surgery**												*0.746*
	< 65 years	58	53	148	56	61	62	22	61	6	60	
	≥ 65 years	51	47	118	44	38	38	14	39	4	40	
**Karnofsky performance status scale**												*0.404*
	≥ 80%	100	92	241	91	92	93	35	97	8	80	
	< 80%	9	8	25	9	7	7	1	3	2	20	
**Tumor stage**												***0.019***
	pT1/2	72	66	177	67	70	71	17	47	3	30	
	pT3/4	37	34	89	33	29	29	19	53	7	70	
**Regional lymph node metastasis**												***0.003***
	N0/pN0	103	95	250	94	93	94	28	78	7	70	
	pN+	6	5	16	6	6	6	8	22	3	30	
**Distant metastasis**												***0.005***
	M0	94	86	231	87	78	79	24	67	6	60	
	M+	15	14	35	13	21	21	12	33	4	40	
**Fuhrman grade**												*** < 0.0001***
	G1/G2	91	83	224	85	78	80	20	56	4	40	
	G3/G4	18	17	39	15	19	20	16	44	6	60	
**Histopathological subtype**												< 0.001**
	Clear cell RCC	88	81	226	85	71	72	31	86	6	60	
	Papillary (chromophilic) RCC	7	6	27	10	17	17	3	8	1	10	
	Chromophobe RCC	11	10	8	3	4	4	0	0	0	0	
	Duct Bellini	0	0	0	0	0	0	2	6	1	10	
	Unclassified RCC	3	3	5	2	7	7	0	0	2	20	

**Motzer criteria (metastatic RCC, n = 51)**												0.731*
	Favorable	1	11	1	6	0	0	2	20	0	0	
	Intermediate	6	67	11	61	6	55	5	50	3	100	
	Poor	2	22	6	33	5	45	3	30	0	0	

Fisher's exact test* using Monte Carlo Simulation**

**Figure 2:**
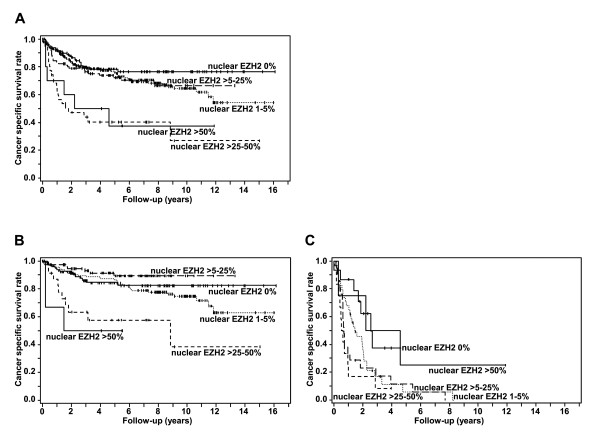
**Kaplan Meier survival curves of cancer specific survival (CSS)**. **A** CSS rates of RCC patients with non-metastatic and metastatic disease. **B **CSS rates in non-metastasized RCC. **C **CSS rates in metastatic RCC.

As shown in Figures 2A and 2B, Kaplan Meier cancer specific survival curves of the whole cohort of patients and of the non-metastatic subgroup revealed a worse prognosis in patients with more than 25% nuclear EZH2 staining. In patients with metastatic disease, nuclear EZH2 expression above 5% and up to 50% was an independent predictor of poor CSS (Figure 2C).

On univariate survival analyses, the risk of death from non-metastasized RCC for patients with high EZH2 positive nuclei (> 25%) was enhanced above that for RCC patients who had no (0%) nuclear EZH2 staining. Apart from EZH2, the following clinical and histopathological features showed a statistically significant impact on CSS in non-metastasized RCC patients in univariate analyses: tumor stage, grading, Karnofsky performance status, and histopathological subtype (Table 3). In metastasized RCCs, the risk of death for patients with EZH2 positive nuclei (> 5% up to 50%) was clearly enhanced above that for RCC patients who had no (0%) nuclear EZH2 staining in the tumor. In patients who had very high (> 50%) nuclear EZH2 staining, only a negative tendency but no statistically significant impact on CSS was seen, possibly due to the small number of cases investigated for this group. The same findings were observed for multivariate analyses, as discussed further below. No other of the investigated clinical or histopathological features showed a statistically significant impact on CSS in univariate analyses (Table 4).

**Table 3:** Analysis of cancer specific survival (CSS) in RCC, patients without metastases

	Univariate Analysis			Multivariate Analysis		
Variable	HR	95% CI	*p*	HR	95% CI	*p*
Stage II vs. I	4.32	2.13-8.73	*** < 0.0001***	3.11	1.48-6.54	***0.003***
Stage III vs. I	8.94	5.35-14.93	*** < 0.0001***	4.84	2.66-8.80	*** < 0.0001***
Grading 2 vs. 1	1.42	0.73-2.75	*0.302*	1.11	0.55-2.23	*0.768*
Grading 3/4 vs. 1	8.91	4.51-17.59	*** < 0.0001***	2.74	1.26-5.95	***0.011***
Karnofsky < 80 vs. ≥ 80	3.02	1.66-5.48	***0.0003***	1.82	0.95-3.51	*0.072*
Age ≥ 65 years vs. < 65 years	1.50	0.95-2.35	*0.081*	1.01	0.62-1.65	*0.965*
Sex (Male vs. Female)	1.16	0.73-1.84	*0.527*	1.37	0.83-2.24	*0.216*
Clear cell RCC vs. non clear cell RCC	5.43	1.71-17.24	***0.004***	2.82	0.87-9.22	*0.086*
Nuclear EZH2 1-5% vs. 0%	1.16	0.64-2.10	*0.615*	1.21	0.66-2.21	*0.546*
Nuclear EZH2 >5-25% vs. 0%	0.57	0.23-1.39	*0.215*	0.60	0.22-1.59	*0.301*
Nuclear EZH2 >25-50% vs. 0%	3.44	1.54-7.67	***0.003***	2.72	1.14-6.50	***0.025***
Nuclear EZH2 >50% vs. 0%	5.11	1.47-17.76	***0.010***	2.20	0.28-17.42	*0.456*

**Table 4:** Analysis of cancer specific survival (CSS) in RCC, patients with metastases

	Univariate Analysis			Multivariate Analysis		
Variable	HR	95% CI	*p*	HR	95% CI	*p*
Grading 2 vs. 1	0.59	0.24-1.44	*0.247*	0.65	0.25-1.74	*0.395*
Grading 3/4 vs. 1	1.03	0.43-2.45	*0.950*	1.18	0.47-3.01	*0.725*
Karnofsky < 80 vs. ≥ 80	1.90	0.99-3.64	*0.054*	2.24	0.99-5.05	*0.052*
Age ≥ 65 years vs. < 65 years	1.39	0.84-2.31	*0.202*	1.47	0.79-2.73	*0.228*
Sex (Male vs. Female)	1.28	0.75-2.19	*0.372*	1.35	0.74-2.45	*0.331*
Clear cell RCC vs. non clear cell RCC	0.59	0.25-1.38	*0.223*	0.36	0.15-0.88	***0.025***
Nuclear EZH2 1-5% vs. 0%	2.18	0.95-5.00	*0.065*	2.63	1.03-6.71	***0.043***
Nuclear EZH2 >5-25% vs. 0%	2.92	1.22-6.97	***0.016***	3.35	1.29-8.74	***0.013***
Nuclear EZH2 >25-50% vs. 0%	3.91	1.51-10.16	***0.005***	4.92	1.72-14.10	***0.003***
Nuclear EZH2 >50% vs. 0%	0.98	0.25-3.91	*0.978*	0.70	0.13-3.75	*0.677*

Next, we investigated whether EZH2 may also independently correlate with CSS in RCC. Multivariate Cox regression analyses on RCC outcome included tumor stage, Fuhrman's grading, Karnofsky performance status, age, sex, histopathological subtype, and EZH2 expression. These analyses revealed that >25-50% nuclear EZH2 expression in the tumor significantly correlated with an increased risk of cancer specific death in patients suffering from non-metastasized RCC (HR 2.72, p = 0.025) (Table 3). Apart from EZH2, tumor stage and high Fuhrman's grading (3/4 vs. 1) emerged as significant prognostic indicators, whereas sex, Karnofsky performance status, age, and histopathological subtype did not independently predict the clinical outcome. For metastasized RCC, 1-5%, >5-25%, and >25-50% nuclear EZH2 expression was linked to decreased CSS when compared with tumors with undetectable EZH2 expression (HR 2.63, p = 0.043, HR 3.35, p = 0.013, HR 4.92, p = 0.003). For the group of very high (> 50%) EZH2 expression no significant influence on survival could be shown in comparison to the group with non-expression of EZH2. Tumor stage, grading, Karnofsky performance status, age, and sex did not predict clinical outcome, whereas clear cell histology showed a positive correlation with CSS (HR 0.36, p = 0.025) (Table 4). In a subgroup of patients with metastatic RCC (n = 51), we included EZH2 expression and the Motzer criteria as the only two factors in a multivariate cox proportional hazards model, which reviewed EZH2 as a borderline significant factor, despite the small number of patients in the analyses (data not shown).

Finally, for further evaluation of the predictive value of EZH2 expression, the concordance probability of the Cox regression models including or excluding EZH2 was calculated. In RCC patients without metastases, the concordance probability of the Cox regression models including the EZH2 status was 73.4%, compared to 71.8% in models excluding the EZH2 status but retaining all other variables. In patients with RCC metastatic disease, the concordance probability including EZH2 expression was 68.4%, compared to 63.0% in models excluding EZH2 expression.

## Discussion

The present study defines EZH2 as a powerful and independent negative prognostic marker of CSS in patients with metastasized and non-metastasized RCC. Thus, assessment of EZH2 expression may allow improved patient selection for systemic therapies. Moreover, integration of the EZH2 status into current prognostic models could result in more accurate survival prediction and may also be useful for individualizing follow-up and selecting patients for clinical trials.

RCC is commonly characterized by a poor response towards current treatment options. Even after complete resection of the primary tumor, relapse occurs in 20-30% of cases. The overall 5-year survival rate is 60%; in patients with metastases, the median survival is only about 13 months, with a 5-year survival of less than 10% [23,24]. Cytokine therapy and novel targeted therapies, i.e. tyrosine kinase inhibitors, have been of limited benefit [23,25-29]. Progression and treatment response of the disease are still not sufficiently predictable. Suitable molecular markers could help to refine individual risk stratification and treatment plans [30]. However, for RCC, as for other cancers in general, only few markers have been validated for clinical practice. This may be partly due to the fact that many studies have been small and poorly designed, used inappropriate statistical analysis, and employed different assay methods and outcome measures [31].

The present study, defining EZH2 as a novel prognostic marker in RCC, has been conducted according to the REMARK criteria [21]. These include a large sample size, a long prospective follow-up of patients, and a description of the predictive value of the marker. Possible limitations of our study include the lack of external validation using a second cohort of patients with RCC. For analyzing RCC patients without metastases, our Cox regression model-which included tumor stage, grading, Karnofsky performance index, age, sex, histopathological subtype, and EZH2 expression-revealed a concordance probability of 73.4% compared to 71.8% excluding EZH2 expression. In RCC patients with metastatic disease, the concordance probability including EZH2 expression was 68.4%, compared to 63.0% excluding EZH2 expression. These values are in a similar range as obtained by the University of California Los Angeles Integrated Staging System (UISS) [32], a well-accepted prognostic factor model for RCC with good predictive accuracy, which exhibits a concordance index of 76% [32]. Including molecular markers to the clinical models increased the concordance probability, in our model as well as in the UISS model [33,34]. Therefore, EZH2 should represent a powerful new marker for predicting prognosis in RCC and could be integrated in established prognostic models to provide an even better consultation for patients regarding diagnosis, treatment, and follow-up.

Our findings concerning the prognostic value of EZH2 in RCC, are in contrast to a recent study of 119 clear cell carcinoma patients, who reported that high tumor EZH2 levels indicate less aggressive tumor phenotypes with a favorable prognosis, as assessed by real-time RT-PCR [35]. We do not know whether this discrepancy is related to the different detection method for EZH2 expression. Our results are in line with studies in several other tumor forms, including malignant melanoma and cancers of the breast, prostate, endometrium, stomach, and liver, where increased EZH2 expression has been linked to more aggressive tumor behaviour and poor prognosis [7,9,17,36,37]. EZH2 expression showed significant prognostic impact in melanoma, prostate, and endometrial carcinoma in univariate survival analyses, but revealed independent multivariate prognostic importance only in carcinoma of the endometrium and prostate [17]. In breast cancer, high EZH2 expression was a strong independent predictive parameter of outcome, providing a better information about CSS than other independent prognostic features [7]. Thus, *EZH2 *may be an interesting novel prognostic marker for a large panel of different cancer types.

RCCs exhibited significantly higher EZH2 expression levels than histologically normal kidney, indicating that an increase in EZH2 expression is acquired during RCC tumorigenesis. Increased EZH2 expression in tumorous versus corresponding normal tissue has been also reported for other cancers as well, including malignant melanoma, prostate carcinoma, breast cancer and hepatocellular carcinoma [7,9,17,37]. However, it should be noted that infiltrating lymphocytes and, sporadically, proximal and distal tubule epithelial cells stained positive for EZH2 in normal renal tissue. This indicates that detectable EZH2 expression is not stringently restricted to tumor cells. In line, EZH2 expression could be detected in the proliferating parabasal cell layer in normal cervical epithelium [14] and in proliferating cells of normal mammary gland tissue [38]. Interestingly, the latter study raised the possibility that EZH2 is expressed in mammary stem cells, in line with studies indicating a dual role of the PcG proteins in self-renewal of stem cells and oncogenesis [39,40].

Apart form serving as a novel prognostic marker in RCC, EZH2 expression may also have therapeutic and diagnostic implications. Mechanistically, *EZH2 *is likely to contribute to the growth of RCC cells, since silencing of *EZH2 *expression exerts profound anti-proliferative effects in RCC lines [13]. These findings indicate that *EZH2 *may represent a novel therapeutic target for RCC treatment in that specific *EZH2 *inhibitors should repress tumor growth. Under diagnostic aspects, it is noteworthy that upregulation of EZH2 expression can be detected very early in breast cancer development, even before atypic cells are histologically evident [15,16]. Thus, the determination of EZH2 expression may be an important new tool to identify patients at risk for developing breast cancer [15,16]. In view of the substantial portion of RCCs expressing EZH2, it will be interesting to investigate in future studies whether EZH2 expression is an early event in RCC development and may also have diagnostic potential for RCC detection.

## Conclusions

We here identified EZH2 expression as a novel, powerful, and independent unfavourable prognostic marker for CSS in patients with both metastatic and non-metastatic RCC. Assessment of the EZH2 status could therefore be integrated in established prognostic models in order to improve clinical management of RCC patients. The high proportion of RCCs showing increased EZH2 protein levels may also have therapeutic implications, since targeted inhibition of EZH2 expression has been shown to repress tumor cell growth.

## Competing interests

The authors declare that they have no competing interests.

## Authors' contributions

NW carried out the conception and the design of the study, participated in the acquisition, analysis and interpretation of data and drafted the manuscript. SMG carried out the generation of the tissue micro arrays and participated in the immunohistochemistry and data analysis. MP participated in the design of the study and performed the statistical analysis. JH helped with the design of the study and participated in statistical analysis. KHS conceived of the study, and participated in its design and coordination and helped to draft the manuscript. PS and JP participated in the design and coordination of the study. AH helped with the conception of the study, the statistical analysis and the critical revision of the manuscript. FHS participated in the design and conception of the study and interpretation of data and helped to draft the manuscript. MH participated in the study design and revised the manuscript critically for important intellectual content. All authors read and approved the final manuscript.

## Pre-publication history

The pre-publication history for this paper can be accessed here:

http://www.biomedcentral.com/1471-2407/10/524/prepub

## References

[B1] Jemal A Siegel R Ward E Hao Y Xu J Thun MJ Cancer statistics, 2009 CA Cancer J Clin 2009 59 4 225 249 10.3322/caac.20006 19474385

[B2] Baldewijns MM van Vlodrop IJ Schouten LJ Soetekouw PM de Bruine AP van Engeland M Genetics and epigenetics of renal cell cancer Biochim Biophys Acta 2008 1785 2 133 155 18187049 10.1016/j.bbcan.2007.12.002

[B3] Cao R Wang L Wang H Xia L Erdjument-Bromage H Tempst P Jones RS Zhang Y Role of histone H3 lysine 27 methylation in Polycomb-group silencing Science 2002 298 5595 1039 1043 10.1126/science.1076997 12351676

[B4] Czermin B Melfi R McCabe D Seitz V Imhof A Pirrotta V Drosophila enhancer of Zeste/ESC complexes have a histone H3 methyltransferase activity that marks chromosomal Polycomb sites Cell 2002 111 2 185 196 10.1016/S0092-8674(02)00975-3 12408863

[B5] Vire E Brenner C Deplus R Blanchon L Fraga M Didelot C Morey L Van Eynde A Bernard D Vanderwinden JM Bollen M Esteller M Di Croce L de Launoit Y Fuks F The Polycomb group protein EZH2 directly controls DNA methylation Nature 2006 439 7078 871 874 10.1038/nature04431 16357870

[B6] Bracken AP Pasini D Capra M Prosperini E Colli E Helin K EZH2 is downstream of the pRB-E2F pathway, essential for proliferation and amplified in cancer Embo J 2003 22 20 5323 5335 10.1093/emboj/cdg542 14532106 PMC213796

[B7] Kleer CG Cao Q Varambally S Shen R Ota I Tomlins SA Ghosh D Sewalt RG Otte AP Hayes DF Sabel MS Livant D Weiss SJ Rubin MA Chinnaiyan AM EZH2 is a marker of aggressive breast cancer and promotes neoplastic transformation of breast epithelial cells Proc Natl Acad Sci USA 2003 100 20 11606 11611 10.1073/pnas.1933744100 14500907 PMC208805

[B8] Croonquist PA Van Ness B The polycomb group protein enhancer of zeste homolog 2 (EZH 2) is an oncogene that influences myeloma cell growth and the mutant ras phenotype Oncogene 2005 24 41 6269 6280 10.1038/sj.onc.1208771 16007202

[B9] Varambally S Dhanasekaran SM Zhou M Barrette TR Kumar-Sinha C Sanda MG Ghosh D Pienta KJ Sewalt RG Otte AP Rubin MA Chinnaiyan AM The polycomb group protein EZH2 is involved in progression of prostate cancer Nature 2002 419 6907 624 629 10.1038/nature01075 12374981

[B10] Tang X Milyavsky M Shats I Erez N Goldfinger N Rotter V Activated p53 suppresses the histone methyltransferase EZH2 gene Oncogene 2004 23 34 5759 5769 10.1038/sj.onc.1207706 15208672

[B11] Berezovska OP Glinskii AB Yang Z Li XM Hoffman RM Glinsky GV Essential role for activation of the Polycomb group (PcG) protein chromatin silencing pathway in metastatic prostate cancer Cell Cycle 2006 5 16 1886 1901 16963837 10.4161/cc.5.16.3222

[B12] Tan J Yang X Zhuang L Jiang X Chen W Lee PL Karuturi RK Tan PB Liu ET Yu Q Pharmacologic disruption of Polycomb-repressive complex 2-mediated gene repression selectively induces apoptosis in cancer cells Genes Dev 2007 21 9 1050 1063 10.1101/gad.1524107 17437993 PMC1855231

[B13] Wagener N Holland D Bulkescher J Crnkovic-Mertens I Hoppe-Seyler K Zentgraf H Pritsch M Buse S Pfitzenmaier J Haferkamp A Hohenfellner M Hoppe-Seyler F The enhancer of zeste homolog 2 gene contributes to cell proliferation and apoptosis resistance in renal cell carcinoma cells Int J Cancer 2008 123 7 1545 1550 10.1002/ijc.23683 18623083

[B14] Holland D Hoppe-Seyler K Schuller B Lohrey C Maroldt J Durst M Hoppe-Seyler F Activation of the enhancer of zeste homologue 2 gene by the human papillomavirus E7 oncoprotein Cancer Res 2008 68 23 9964 9972 10.1158/0008-5472.CAN-08-1134 19047178

[B15] Ding L Erdmann C Chinnaiyan AM Merajver SD Kleer CG Identification of EZH2 as a molecular marker for a precancerous state in morphologically normal breast tissues Cancer Res 2006 66 8 4095 4099 10.1158/0008-5472.CAN-05-4300 16618729

[B16] Ding L Kleer CG Enhancer of zeste 2 as a marker of preneoplastic progression in the breast Cancer Res 2006 66 19 9352 9355 10.1158/0008-5472.CAN-06-2384 17018586

[B17] Bachmann IM Halvorsen OJ Collett K Stefansson IM Straume O Haukaas SA Salvesen HB Otte AP Akslen LA EZH2 expression is associated with high proliferation rate and aggressive tumor subgroups in cutaneous melanoma and cancers of the endometrium, prostate, and breast J Clin Oncol 2006 24 2 268 273 10.1200/JCO.2005.01.5180 16330673

[B18] Motzer RJ Bacik J Schwartz LH Reuter V Russo P Marion S Mazumdar M Prognostic factors for survival in previously treated patients with metastatic renal cell carcinoma J Clin Oncol 2004 22 3 454 463 10.1200/JCO.2004.06.132 14752067

[B19] Macher-Goeppinger S Aulmann S Wagener N Funke B Tagscherer KE Haferkamp A Hohenfellner M Kim S Autschbach F Schirmacher P Roth W Decoy receptor 3 is a prognostic factor in renal cell cancer Neoplasia 2008 10 10 1049 1056 18813347 10.1593/neo.08626 PMC2546583

[B20] Dukers DF van Galen JC Giroth C Jansen P Sewalt RG Otte AP Kluin-Nelemans HC Meijer CJ Raaphorst FM Unique polycomb gene expression pattern in Hodgkin's lymphoma and Hodgkin's lymphoma-derived cell lines Am J Pathol 2004 164 3 873 881 14982841 10.1016/S0002-9440(10)63175-6 PMC1613333

[B21] McShane LM Altman DG Sauerbrei W Taube SE Gion M Clark GM REporting recommendations for tumor MARKer prognostic studies (REMARK) Breast Cancer Res Treat 2006 100 2 229 235 10.1007/s10549-006-9242-8 16932852

[B22] Goenen M Heller G Concordance probability and discriminatory power in proportional hazards regression Biometrika 2005 92 4 965 970 10.1093/biomet/92.4.965

[B23] Cohen HT McGovern FJ Renal-cell carcinoma N Engl J Med 2005 353 23 2477 2490 10.1056/NEJMra043172 16339096

[B24] Chow WH Devesa SS Warren JL Fraumeni JF Jr Rising incidence of renal cell cancer in the United States Jama 1999 281 17 1628 1631 10.1001/jama.281.17.1628 10235157

[B25] Escudier B Eisen T Stadler WM Szczylik C Oudard S Siebels M Negrier S Chevreau C Solska E Desai AA Rolland F Demkow T Hutson TE Gore M Freeman S Schwartz B Shan M Simantov R Bukowski RM Sorafenib in advanced clear-cell renal-cell carcinoma N Engl J Med 2007 356 2 125 134 10.1056/NEJMoa060655 17215530

[B26] Fisher RI Rosenberg SA Fyfe G Long-term survival update for high-dose recombinant interleukin-2 in patients with renal cell carcinoma Cancer J Sci Am 2000 6 Suppl 1 55 57 10685660

[B27] Motzer RJ Hutson TE Tomczak P Michaelson MD Bukowski RM Rixe O Oudard S Negrier S Szczylik C Kim ST Chen I Bycott PW Baum CM Figlin RA Sunitinib versus interferon alfa in metastatic renal-cell carcinoma N Engl J Med 2007 356 2 115 124 10.1056/NEJMoa065044 17215529

[B28] Escudier B Pluzanska A Koralewski P Ravaud A Bracarda S Szczylik C Chevreau C Filipek M Melichar B Bajetta E Gorbunova V Bay JO Bodrogi I Jagiello-Gruszfeld A Moore N Bevacizumab plus interferon alfa-2a for treatment of metastatic renal cell carcinoma: a randomised, double-blind phase III trial Lancet 2007 370 9605 2103 2111 10.1016/S0140-6736(07)61904-7 18156031

[B29] Motzer RJ Escudier B Oudard S Hutson TE Porta C Bracarda S Grunwald V Thompson JA Figlin RA Hollaender N Urbanowitz G Berg WJ Kay A Lebwohl D Ravaud A Efficacy of everolimus in advanced renal cell carcinoma: a double-blind, randomised, placebo-controlled phase III trial Lancet 2008 372 9637 449 456 10.1016/S0140-6736(08)61039-9 18653228

[B30] Eichelberg C Junker K Ljungberg B Moch H Diagnostic and Prognostic Molecular Markers for Renal Cell Carcinoma: A Critical Appraisal of the Current State of Research and Clinical Applicability Eur Urol 2009 55 4 851 863 10.1016/j.eururo.2009.01.003 19155123

[B31] McShane LM Altman DG Sauerbrei W Identification of clinically useful cancer prognostic factors: what are we missing? J Natl Cancer Inst 2005 97 14 1023 1025 10.1093/jnci/dji193 16030294

[B32] Zisman A Pantuck AJ Wieder J Chao DH Dorey F Said JW deKernion JB Figlin RA Belldegrun AS Risk group assessment and clinical outcome algorithm to predict the natural history of patients with surgically resected renal cell carcinoma J Clin Oncol 2002 20 23 4559 4566 10.1200/JCO.2002.05.111 12454113

[B33] Kim HL Seligson D Liu X Janzen N Bui MH Yu H Shi T Belldegrun AS Horvath S Figlin RA Using tumor markers to predict the survival of patients with metastatic renal cell carcinoma J Urol 2005 173 5 1496 1501 10.1097/01.ju.0000154351.37249.f0 15821467

[B34] Kim HL Seligson D Liu X Janzen N Bui MH Yu H Shi T Figlin RA Horvath S Belldegrun AS Using protein expressions to predict survival in clear cell renal carcinoma Clin Cancer Res 2004 10 16 5464 5471 10.1158/1078-0432.CCR-04-0488 15328185

[B35] Hinz S Weikert S Magheli A Hoffmann M Engers R Miller K Kempkensteffen C Expression profile of the Polycomb group protein Enhancer of zeste homologue 2 and its prognostic relevance in renal cell carcinoma J Urol 2009 182 6 2920 2925 10.1016/j.juro.2009.08.014 19846140

[B36] Matsukawa Y Semba S Kato H Ito A Yanagihara K Yokozaki H Expression of the enhancer of zeste homolog 2 is correlated with poor prognosis in human gastric cancer Cancer Sci 2006 97 6 484 491 10.1111/j.1349-7006.2006.00203.x 16734726 PMC11159019

[B37] Sudo T Utsunomiya T Mimori K Nagahara H Ogawa K Inoue H Wakiyama S Fujita H Shirouzu K Mori M Clinicopathological significance of EZH2 mRNA expression in patients with hepatocellular carcinoma Br J Cancer 2005 92 9 1754 1758 10.1038/sj.bjc.6602531 15856046 PMC2362028

[B38] Pietersen AM Horlings HM Hauptmann M Langerod A Ajouaou A Cornelissen-Steijger P Wessels LF Jonkers J van de Vijver MJ van Lohuizen M EZH2 and BMI1 inversely correlate with prognosis and TP53 mutation in breast cancer Breast Cancer Res 2008 10 6 R109 10.1186/bcr2214 19099573 PMC2656906

[B39] Metsuyanim S Pode-Shakked N Schmidt-Ott KM Keshet G Rechavi G Blumental D Dekel B Accumulation of malignant renal stem cells is associated with epigenetic changes in normal renal progenitor genes Stem Cells 2008 26 7 1808 1817 10.1634/stemcells.2007-0322 18467665

[B40] Yu J Yu J Rhodes DR Tomlins SA Cao X Chen G Mehra R Wang X Ghosh D Shah RB Varambally S Pienta KJ Chinnaiyan AM A polycomb repression signature in metastatic prostate cancer predicts cancer outcome Cancer Res 2007 67 22 10657 10663 10.1158/0008-5472.CAN-07-2498 18006806

